# A novel approach to assessing bisphenol-A hazards using an *in vitro* model system

**DOI:** 10.1186/s12864-016-2979-5

**Published:** 2016-08-09

**Authors:** Md Saidur Rahman, Woo-Sung Kwon, Sung-Jae Yoon, Yoo-Jin Park, Buom-Yong Ryu, Myung-Geol Pang

**Affiliations:** Department of Animal Science and Technology, Chung-Ang University, Anseong, Gyeonggi-Do 456-756 Republic of Korea

**Keywords:** Bisphenol-A, Health hazards, *In-vitro*, Spermatozoa, Proteomics, Bioinformatics databases, Diseases, Signaling pathways

## Abstract

**Background:**

Although the toxicological impacts of the xenoestrogen bisphenol-A (BPA) have been studied extensively, but the mechanism of action is poorly understood. Eventually, no standard method exists for evaluating the possible health hazards of BPA exposure. Considering mice spermatozoa as a potential *in vitro* model, we investigated the effects of BPA exposure (0.0001, 0.01, 1, and 100 μM for 6 h) on spermatozoa and the related mechanisms of action. The same doses were also employed to evaluate protein profiles of spermatozoa as a means to monitor their functional affiliation to diseases.

**Results:**

Our results demonstrated that high concentrations of BPA negatively affect sperm motility, viability, mitochondrial functions, and intracellular ATP levels by activating the mitogen-activated protein kinase, phosphatidylinositol 3-kinase, and protein kinase-A pathways. Moreover, short-term exposure of spermatozoa to high concentrations of BPA induced differential expressions of 24 proteins. These effects appeared to be caused by protein degradation and phosphorylation in spermatozoa. Proteins differentially expressed in spermatozoa from BPA treatment groups are putatively involved in the pathogenesis of several diseases, mainly cancer, carcinoma, neoplasm, and infertility.

**Conclusions:**

Based on these results, we propose that BPA adversely affects sperm function by the activation of several kinase pathways in spermatozoa. In addition, BPA-induced changes in the sperm proteome might be partly responsible for the observed effects in spermatozoa, subsequently involve in the pathogenesis of many diseases. Therefore, we anticipated that current strategy might broadly consider for the health hazards assessment of other toxicological agents.

**Electronic supplementary material:**

The online version of this article (doi:10.1186/s12864-016-2979-5) contains supplementary material, which is available to authorized users.

## Background

Bisphenol-A (BPA) is an estrogenic endocrine disruptor and is produced annually in large quantities for the production of polycarbonate plastics and epoxy resins. Because of hormone-like properties, concerns have been raised about the sustainability of BPA in consumer products. The Centers for Disease Control and Prevention has identified high levels of urinary BPA in >90 % of the U.S. population [1]. BPA is associated with impaired reproductive functions as well as other diseases, such as cancer, diabetes, obesity, cardiovascular disease, thyroid dysfunction, developmental disorders, and miscarriages [2]. Several *in vivo* studies have reported that BPA-exposed males are prone to reproductive developmental disorders [3, 4] and that the spermatozoa of exposed males are incapable of proper sperm-oocyte binding, thereby affecting embryonic growth [5]. Recently we have reported that BPA-induced modifications of fertility-related proteins in spermatozoa are associated with increased reproductive health risk [6].

Genomic and non-genomic pathways are two proposed mechanisms by which xenoestrogens exert their hormone-like action. In the genomic pathway, xenoestrogens trigger estrogen receptors (ERs, nuclear and cytoplasmic) and lead to the transcription of target genes, thereby affecting cell function [7]. In the non-genomic pathway, G protein-coupled receptors (GPCRs) together with membrane-bound ERs (non-genomic) cause rapid estrogenic signaling through activation of mitogen-activated protein kinase (MAPK) and phosphatidylinositol 3-kinase (PI3K), changes in cAMP/protein kinase-C (PKC), and fluctuation of intracellular calcium [8]. Consistently, BPA also has been reported to exert its effects via activation of MAPK and PI3K in human ovarian cancer cells [9, 10]. As demonstrated, mature spermatozoa contain functional GPCRs [11] and non-genomic ERs [12], therefore, provide a valid model to investigate the molecular mechanism of BPA action [6, 13, 14].

Recent advances in proteomic research have offered a new vista for developing proteomic biomarkers of chemical exposure, with the aim of evaluating hostile health effects [15]. In particular, two-dimensional gel electrophoresis (2-DE) coupled with mass spectrometry (MS) has been used effectively in several toxicology studies and in high-throughput industrial applications. A direct comparison of protein expression levels from control versus altered conditions reveals a set of biomarkers representative of the altered state. Since spermatozoa have no functional protein synthesizing machinery, therefore provide a valid model for proteomic analysis [16–18]. Consequently, several laboratories have applied this technique to develop diagnostic biomarkers of spermatozoa in different functional states [16, 17]. However, to date, efforts to establish protein expression profiles indicative of xenoestrogen (BPA) exposure in spermatozoa have not been attempted. Therefore, the objectives of the current study were: 1) to determine whether different concentrations of BPA could affect sperm functions, and if so, to investigate the mechanisms linked with these observed effects and 2) to identify the differentially expressed proteins of exposure as a manner to screen their functional affiliation to diseases.

## Results

### The effect of BPA on motility, viability, and intracellular lactate dehydrogenase (LDH) levels in spermatozoa

**Fig. 1:**
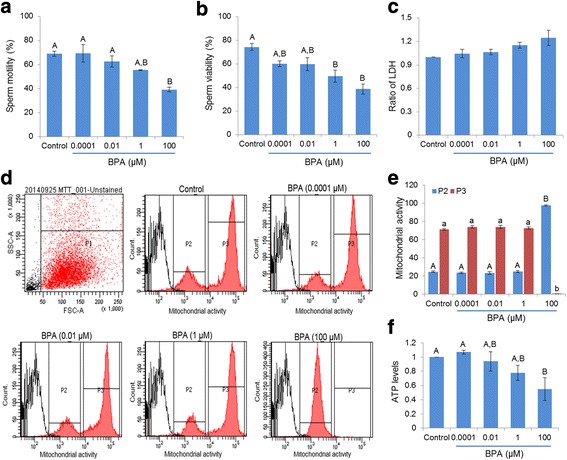
Effect of BPA on motility, viability, lactate dehydrogenase (LDH), mitochondrial activity, and intracellular ATP ([ATP]_i_) levels. (**a**) Differences in motility in BPA-treated and control spermatozoa. (**b**) Differences in number of viable spermatozoa between the control and BPA treatment. (**c**) Differences in LDH levels in control and BPA-treated spermatozoa. (**d**) Representative flow cytometry images of mitochondrial activity in control and BPA-treated spermatozoa. (**e**) The bar represents the difference in mitochondrial activity detected by flow cytometry in control and BPA-treated spermatozoa. (**f**) Differences in [ATP]_i_ levels between control and BPA-treated spermatozoa. Data are presented as mean ± SEM (4 replicates). Values with different superscript characters (^A,B,a,b^) indicate significant differences between the control and treatment groups as determined by one-way ANOVA (*p* < 0.05)

The percentage of motile spermatozoa decreased significantly in the presence of 100 μM of BPA (*p* < 0.05) (Fig. 1a). However, the sperm viability was affected significantly by exposure to both 1 and 100 μM BPA (*p* < 0.05) (Fig. 1b). In contrast, a non-significant increase of intracellular LDH levels was noted in all concentrations of BPA tested (Fig. 1c).

### The effect of BPA on mitochondrial activity and intracellular ATP ([ATP]_i_) levels in spermatozoa

Mitochondrial respiration produces ATP, which is required to maintain sperm motility and other biochemical modifications required for fertilization [19]. Current study showed that a high concentration of BPA (100 μM) significantly altered the mitochondrial activity of spermatozoa (*p* < 0.05) (Fig. 1d and e). To validate whether these alterations of mitochondrial activity were correlated with overall [ATP]_i_ production, we measured the [ATP]_i_ levels in spermatozoa. Our results revealed that high concentrations of BPA were also able to significantly reduce [ATP]_i_ levels compared with those of other tested concentrations and the control (*p* < 0.05) (Fig. 1f).

### BPA stimulates MAPK, PI3K, and PKA pathways in spermatozoa

**Fig. 2:**
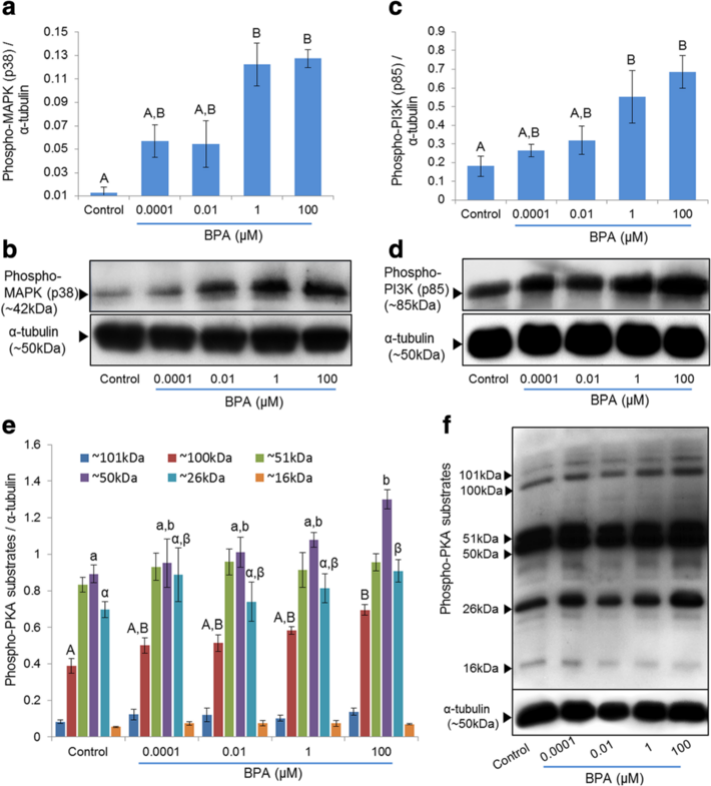
Effect of BPA on MAPK (p38), PI3K (p85), and PKA signalings pathways in spermatozoa. (**a**) The bar represents the densities of phospho-MAPK (p38) in BPA-treated and control spermatozoa. (**b**) Representative western blot image of phospho-MAPK (p38) probed with specific antibody. (**c**) Densities of phospho-PI3K (p85) in BPA-treated and control spermatozoa. (**d**) Representative western blot image of phospho-PI3K (p85) probed with specific antibody. (**e**) Densities of PKA substrate species in BPA-treated and control spermatozoa. (**f**) Phospho-PKA substrates were probed with an anti-phospho-PKA antibody. Lane 1: control; lane 2: 0.0001 μM BPA; lane 3: 0.01 μM BPA; lane 4: 1 μM BPA; and lane 5: 100 μM BPA. Data are presented as mean ± SEM (3 replicates). Values with different superscript characters (^A,B,a,b,α,β^) indicate significant differences between the control and treatment groups as determined by one-way ANOVA (*p* < 0.05)

As reported previously [9, 10], BPA activates the MAPK and PI3K signaling pathways in ovarian cancer cells. To evaluate this possibility in spermatozoa, activation of MAPK(p38) and PI3K(p85) were evaluated using western blot analyses. Our results showed that BPA induced a dose-dependent increase in both phospho-MAPK (p38) and phospho-PI3K (p85) (Fig. 2a–d). Further, it has been demonstrated that the activation of the MAPK/PI3K signaling pathways regulates PKA activity to maintain complex cross-talk between these pathways [20, 21]. Therefore, to further discriminate the PKA activity in spermatozoa due to exposure of BPA, PKA levels were also measured. Significant increases in the levels of three PKA substrates (~100, 50, and 26 kDa) were observed in the presence of 100 μM of BPA (*p* < 0.05) (Fig. 2e and f).

### Identification and quantification of proteins differentially expressed in control and BPA-exposed spermatozoa

**Fig. 3:**
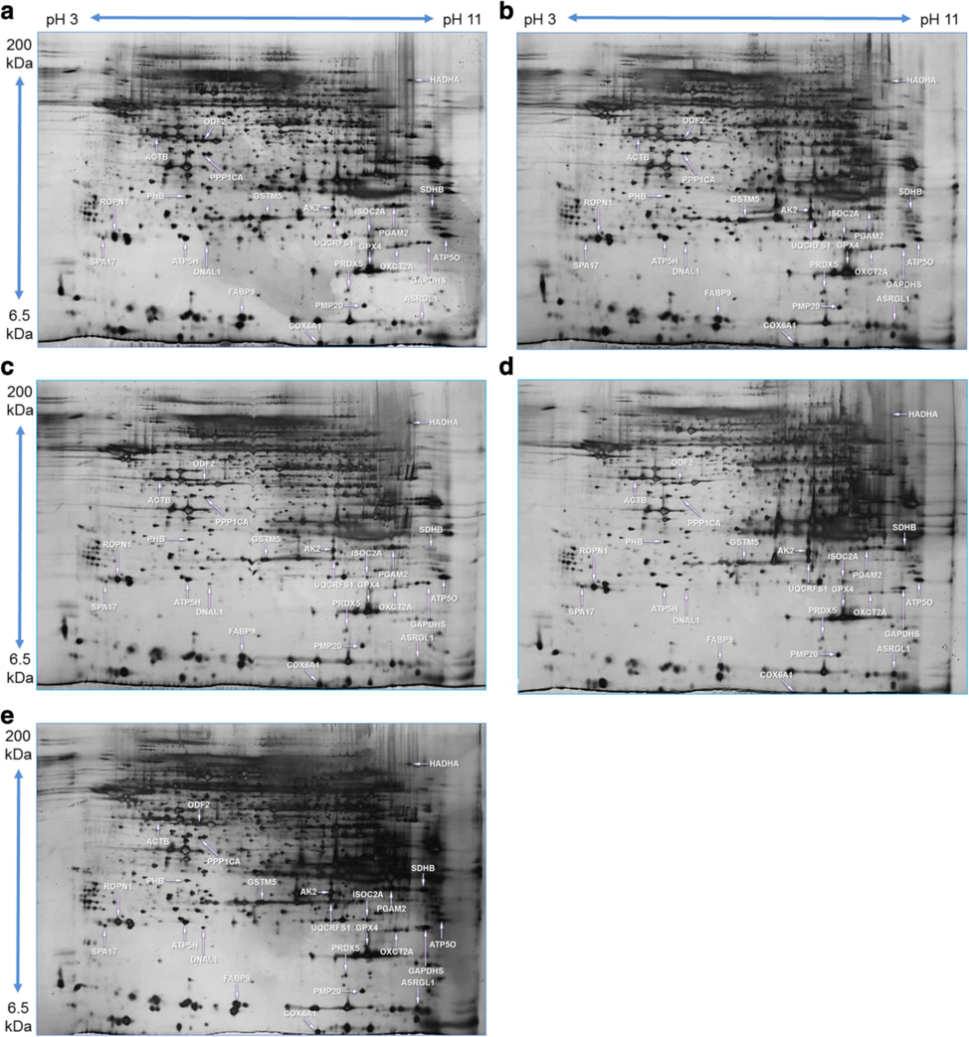
Representative silver nitrate stained image of 2-DE protein spot. (**a**) Protein spots of control spermatozoa. (**b**) Protein spots of 0.0001 μM of BPA-treated spermatozoa. (**c**) Protein spots of 0.01 μM of BPA-treated spermatozoa. (**d**) Protein spots from 1 μM of BPA treated-spermatozoa. **e** Protein spots of 100 μM BPA-treated spermatozoa

To learn pathophysiological importance of BPA exposure, we applied a proteomic approach (2-DE coupled with MS) for identification of differentially expressed proteins in spermatozoa. On average, 399 spots were consistently detected in all gels, 250 of which shared almost similar expression patterns across treatments. Fifty spots showed a dose-dependent expression profile, but significant (*P <* 0.05) changes were noticed in 29 spots. In our assessment of the dose-dependent expression profiles, we avoided nine spots that demonstrated non-linear differences in expression between treated and control groups. Finally, we identified 24 spots by electrospray ionization tandem mass spectrometry (ESI-MS/MS). The identified proteins spots and their normalized spot values in the control and BPA-treated spermatozoa are shown in Fig. 3 and Table 1, respectively (the detailed information regarding peptide sequence, matches peptides, and used searching engine of identified proteins were provided in Additional file 1: Table S1). Our results showed that 24 proteins (16 down- and 9 up-regulated) were differentially expressed between BPA-treated (100 μM) and control spermatozoa (Table 1). The number of differentially expressed proteins in control *vs* 0.01 μM and control *vs* 1 μM were 3 (2 down- and 1 up-regulated) and 7 (4 down- and 3 up-regulated), respectively (Table 1). Additionally, using Gene Ontology annotation in bioinformatics databases (UniProt-GOA and Human Sperm Proteome), the identified proteins were characterized according to their biological function as listed in Table 1.

**Table 1:** Proteins with a significantly lower or higher expression in treatment and control groups

Symbol	Protein description	gi no.	MASCOT score*	Relative intensity^a^ (normalized)				
				Control	0.0001	0.01	1	100
Energy metabolism (46 %)								
ATP5H	ATP synthase subunit d, mitochondrial	gi|21313679	58	1^A^	0.99 ± 0.15^A^	0.93 ± 0.12^A^	0.63 ± 0.01^A,B^	0.36 ± 0.12^B^
ATP5O	ATP synthase subunit O, mitochondrial	gi|20070412	69	1^A^	0.97 ± 0.19^A^	0.68 ± 0.14^A,B^	0.62 ± 0.08^A,B^	0.44 ± 0.07^B^
HADHA	Trifunctional enzyme subunit alpha, mitochondrial	gi|33859811	66	1^A^	0.71 ± 0.01^A,B^	0.41 ± 0.03^B,C^	0.40 ± 0.04^B,C^	0.31 ± 0.10^C^
COX6A1	Cytochrome c oxidase subunit 6A1, mitochondrial	gi|1352173	49	1^A^	0.68 ± 0.19^A,B^	0.28 ± 0.07^B,C^	0.23 ± 0.06^B,C^	0.17 ± 0.01^C^
PGAM2	Phosphoglycerate mutase 2	gi|9256624	53	1^A^	0.99 ± 0.13^A^	0.76 ± 0.13^A,B^	0.72 ± 0.08^A,B^	0.34 ± 0.07^B^
OXCT2A	Succinyl-CoA:3-ketoacid coenzyme A transferase 2A, mitochondrial	gi|81881929	36	1^A^	0.71 ± 0.16^A,B^	0.67 ± 0.07^A,B^	0.65 ± 0.04^A,B^	0.43 ± 0.09^B^
ISOC2A	Isochorismatase domain-containing protein 2A, mitochondrial	gi|197333728	73	1^A^	0.83 ± 0.19^A,B^	0.79 ± 0.08^A,B^	0.75 ± 0.10^A,B^	0.34 ± 0.12^B^
GAPDH	Glyceraldehyde-3-phosphate dehydrogenase, testis-specific	gi|2494630	37	1^A^	1.09 ± 0.14^A^	1.11 ± 0.25^A,B^	1.12 ± 0.31^A,B^	2.17 ± 0.28^B^
AK2	Adenylate kinase 2, mitochondrial isoform	gi|34328230	70	1^A^	1.13 ± 0.18 ^A,B^	1.19 ± 0.27^A,B^	1.37 ± 0.11^A,B^	2.05 ± 0.14^B^
SDHB	Succinate dehydrogenase Ip subunit	gi|34328286	63	1^A^	2.59 ± 0.66^A,B^	3.02 ± 0.29^A,B^	3.32 ± 0.56^B^	3.27 ± 0.38^B^
UQCRFS1	Cytochrome b-c1 complex subunit Rieske, mitochondrial	gi|13385168	62	1^A^	1.56 ± 0.17^A,B^	1.62 ± 0.40^A,B^	1.72 ± 0.32^A,B^	2.16 ± 0.01^B^
Cytoskeletal/structural proteins (25 %)								
ROPN1	Ropporin-1	gi|74227586	202	1^A^	0.88 ± 0.12^A^	0.78 ± 0.10^A,B^	0.71 ± 0.10^A,B^	0.49 ± 0.01^B^
ACTB	Actin, cytoplasmic 1	gi|6671509	51	1^A^	0.93 ± 0.17^A^	0.92 ± 0.16^A^	0.49 ± 0.04^B^	0.47 ± 0.04^B^
FABP9	Fatty acid-binding protein 9	gi|166897974	68	1^A^	0.94 ± 0.05^A^	0.86 ± 0.13^A^	0.68 ± 0.03^A,B^	0.47 ± 0.07^B^
ODF2	Outer dense fiber protein 2	gi|2290719	57	1^A^	0.68 ± 0.14^A,B^	0.62 ± 0.19^A,B^	0.59 ± 0.16^A,B^	0.13 ± 0.01^B^
PMP20	Peroxisomal membrane protein 20	gi|6746357	79	1^A^	1.06 ± 0.05^A^	1.10 ± 0.06^A,B^	1.17 ± 0.22^A,B^	2.11 ± 0.01^B^
ASRGL1	Isoaspartyl peptidase/L-asparaginase	gi|81875980	52	1^A^	1.38 ± 0.12^A,B^	2.09 ± 0.87^A,B^	2.79 ± 0.42^A,B^	3.23 ± 0.36^B^
Fertility Related Proteins (17 %)								
PHB	Prohibitin	gi|6679299	68	1^A^	0.91 ± 0.12^A^	0.81 ± 0.20^A,B^	0.75 ± 0.12^A,B^	0.43 ± 0.05^B^
PPP1CA	Serine/threonine-protein phosphatase PP1-alpha catalytic subunit	gi|49065812	50	1^A^	0.94 ± 0.16^A^	0.60 ± 0.20^A,B^	0.47 ± 0.07^A,B^	0.36 ± 0.05^B^
DNAL1	Dynein light chain 1, axonemal	gi|164607162	67	1^A^	0.74 ± 0.01^A,B^	0.65 ± 0.14 ^A,B^	0.64 ± 0.23^A,B^	0.49 ± 0.13^B^
SPA17	Sperm surface protein Sp17	gi|6755614	58	1^A^	1.11 ± 0.21^A^	1.39 ± 0.81^A^	1.59 ± 0.05^A,B^	2.08 ± 0.17^B^
Stress response proteins/ROS metabolism (12 %)								
GSTM5	Glutathione S-transferase Mu 5	gi|6754086	70	1^A^	0.92 ± 0.26^A,B^	0.67 ± 0.08^A,B^	0.48 ± 0.09^B^	0.32 ± 0.08^B^
GPX4	Glutathione peroxidase	gi|3075477	65	1^A^	1.71 ± 0.08^A,B^	1.87 ± 0.19^B^	2.24 ± 0.24^B^	2.25 ± 0.15^B^
PRDX5	Peroxiredoxin-5, mitochondrial	gi|6755114	73	1^A^	1.28 ± 0.17^A,B^	1.30 ± 0.15^A,B^	2.02 ± 0.11^B^	2.35 ± 0.09^B^

*MASCOT score is −10 log (P), where P is the probability that the observed match is a random event. Individual scores > 30 indicate identity or extensive homology (*p* < 0.05). ^a^Relative spots intensity in control and BPA-treated (μM) spermatozoa. Data are presented as mean ± SEM (3 replicates). Values with different superscript characters (A,B,C) indicate significant differences between the control and treatment groups as determined by one-way ANOVA (*P* < 0.05)

### Functional analysis and signaling pathways of differentially expressed proteins

**Fig. 4:**
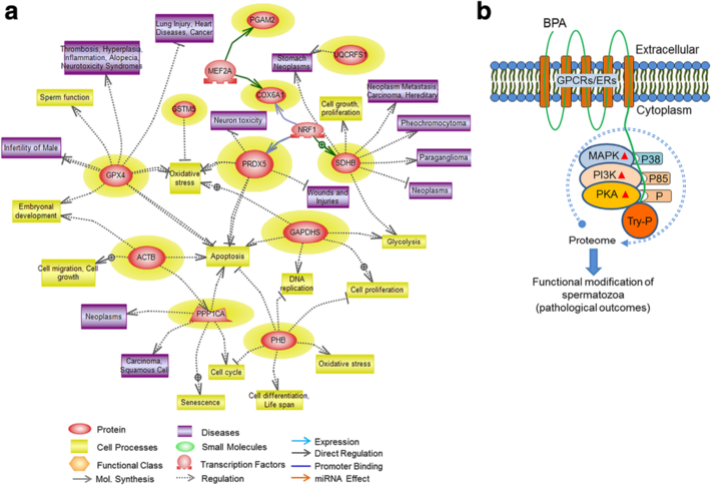
Differentially expressed proteins regulated cellular processes/diseases and hypothetical depiction of BPA action. (**a**) BPA-induced differentially expressed (*p* < 0.05) proteins regulated cellular processes and diseases as depicted by Pathway Studio software. Differentially expressed proteins have been highlighted in yellow background. At least 11 proteins among the 24 differentially expressed proteins were implicated in different disease processes. (**b**) Hypothetical depiction showing the effects of BPA in spermatozoa and the possible mechanism of action. BPA may bind to membrane receptors on spermatozoa that induce rapid phosphorylation of MAPK (p38), PI3K (p85), PKA substrates and subsequent activation of the kinase systems. Simultaneously, BPA induced the phosphorylation of tyrosine residue (Try-P) of sperm proteins. BPA induced changes in spermatozoa affect the overall protein content of the spermatozoa, ultimately predispose to several pathological outcome

Pathway Studio program was used to identify signaling pathways regulated by the differentially expressed proteins. Our results showed a significant association between five canonical signaling pathways and some of the differentially expressed proteins (*p* < 0.05) (Table 2). We also identified and schematized the clinical significance of these proteins using a MedScan Reader (v5.0) search and Pathway Studio, respectively. The disease processes regulated by the differentially expressed proteins are compiled in Fig. 4a.

**Table 2:** Signaling pathways associated with differentially expressed proteins as identified by Pathway Studio program

Signaling Pathways	Overlapping Entities	*P*-value
Respiratory chain and oxidative phosphorylation	UQCRFS1, SDHB, COX6A1, ATP5O	<0.001
Glutathione metabolism	GSTM5, GPX4, PRDX5	0.017
Notch Pathway	ACTB, PPP1CA, HADHA, PGAM2, ODF1, GAPDH	0.017
EphrinR - > actin signaling	ACTB, ODF1	0.030
Adipocytokine Signaling	SDHB, HADHA, PGAM2, GAPDH	0.031

BPA-induced differentially expressed proteins were entered into the Pathway Studio program to identify the significantly signaling pathways. The probabilities of the signaling pathways were determined using the Fisher’s exact test (*p* < 0.05)

### Western blot analysis of six representative proteins and phospho-tyrosine proteins

To validate the 2-DE results, six differentially expressed proteins, such as glyceraldehyde-3-phosphate dehydrogenase (GAPDH), cytochrome b-c1 complex (UQCRFS1), peroxiredoxin-5 (PRDX5), glutathione peroxidase (GPX4), actin, cytoplasmic 1 (ACTB), and glutathione S-transferase (GSTM5) were further verified by western blot analyses using their commercially available antibodies. The western blot results confirmed the differentially expressed protein levels visualized in 2-DE (Additional file 2: Figure S1). Western blot also was applied to investigate the phospho-tyrosine levels (PY20) in both the treated and control spermatozoa. Significant increases in the levels of two tyrosine-phosphorylated species (~100 and ~24 kDa) were detected in BPA-treated (1 and 100 μM) spermatozoa compared with the control (*p* < 0.05) (Additional file 2: Figure S2).

## Discussion

BPA has been regarded as a ubiquitous environmental chemical to which we are exposed in everyday life. Considering the toxicological importance of BPA, here we attempted to investigate whether several concentrations of BPA would affect sperm functions and to investigate the molecular mechanisms. Additionally, BPA was employed to separate differentially expressed proteins in spermatozoa as a manner to monitor their functional affiliation to several disease processes.

Our results showed that higher concentrations of BPA significantly decreased sperm motility (Fig. 1a) and viability (Fig. 1b), even the doses that were potentially non-toxic (Fig. 1c). Several earlier studies also demonstrated the similar action of BPA on sperm motility in rats and mice [6, 22, 23]. It has been reported that mitochondrial respiration mainly provides ATP to support sperm motility [19]. To evaluate this possibility, the effect of BPA on mitochondrial activity and [ATP]_i_ also were evaluated. Consistent with the motility and viability data, 100 μM BPA altered the mitochondrial activity significantly (Fig. 1d and e) and decreased the [ATP]_I_ (Fig. 1f). Collectively, our findings support that BPA (~100 μM) alters the mitochondrial activity and decreases [ATP]_i_ and, thus, could affect sperm functions (motility/viability).

BPA has been demonstrated to activate the MAPK and PI3K signaling pathways in cancer cells [9, 10, 24]. It has been demonstrated that p38 (MAPK) and p85 (PI3K) proteins are rapidly tyrosine-phosphorylated in response to extracellular stimuli and trigger subsequent activation of the kinase systems [25, 26]. We have shown that the addition of BPA to spermatozoa leads to a significant dose-dependent induction of the phospho-MAPK (p38) and phospho-PI3K (p85), thereby activating both kinase pathways (Fig. 2a–d). Activation of MAPK [27] and PI3K [28] also has been reported in ejaculated bovine spermatozoa due to exposure of extracellular stimuli. On the other hand, PKA has been shown to be essential for the regulation of major signaling pathways in spermatozoa [6, 29], as well as cross-talk between MAPK and PI3K signaling [20, 21]. Further, the activation of the PKA pathway by structurally diverse estrogenic compounds, including BPA, also has been reported [24]. Therefore, we performed western blot analysis for PKA activity (Fig. 2e and f), in treated and control spermatozoa. Consistent with previous finding in other cells, collectively our results support that BPA activates the MAPK, PI3K, and PKA signaling in spermatozoa and ultimately affects the cell functions.

A recent technological advance in proteomics is that researchers have successfully developed diagnostic biomarkers of several diseases [15]. Nevertheless, complete proteomic biomarkers indicative of xenoestrogen exposure (eg BPA) in spermatozoa still remain unknown. Therefore, we have performed 2-DE coupled with MS to identify unique protein expression profiles in BPA-treated spermatozoa to predict the pathophysiological consequences of exposure. Our results showed that 24 proteins (16 down- and 9 up-regulated) were dose dependently (*p* < 0.05) expressed between BPA-treated and control spermatozoa (Table 1). Protein degradation (down-regulation) in spermatozoa due to environmental factors is well studied [6, 16, 17]; however, the manner in which protein levels are increased *in vitro* need to be investigated. Mature spermatozoa are silent in both transcription and translation or are poorly capable of translation [6]. Therefore, protein up-regulation could be achieved by posttranslational modification, such as tyrosine phosphorylation [6, 16, 17, 30]. A similar increase in protein tyrosine phosphorylation was also found in spermatozoa due to BPA exposure (Additional file 2: Figure S2). Taken together with several recent studies [6, 16, 17, 30], our results suggest that exposure to BPA induces protein degradation and phosphorylation in spermatozoa, ultimately affecting sperm function.

A total of 11 (46 %) proteins involved in energy metabolism exhibited altered levels in BPA-exposed (100 μM) spermatozoa compared with those of the control (Table 1). Proteins warranting attention for being degraded were mitochondrial ATP synthase subunit d (ATP5H), mitochondrial ATP synthase subunit O (ATP5O), mitochondrial trifunctional enzyme subunit alpha (HADHA), mitochondrial cytochrome c oxidase subunit 6A1 (COX6A1), phosphoglycerate mutase 2 (PGAM2), mitochondrial succinyl-CoA:3-ketoacid coenzyme A transferase 2A (OXCT2A), and mitochondrial isochorismatase domain-containing protein 2A (ISOC2A) (Table 1). Most of the degraded proteins identified in this group were mitochondrial isoforms, thus predominantly involved in energy metabolism. Therefore, down-regulation of these proteins could affect the overall mitochondrial functions and ATP production as also demonstrated in current study (Fig. 1f). Clinically, the ATP synthase subunit and HADHA have been shown to be down-regulated in type 2 diabetes [31] and hepatocellular cancer cells [32], respectively. Moreover, COX6A1 exerts a protective role against ROS-induced cell damage; thus, its down-regulation could lead to abnormal cell functions [33]. It is important to note that a significant down-regulation of HADHA and COX6A1 were detected between the control and 0.01/1 μM BPA exposure (Table 1), and therefore, these proteins could provide potential biomarkers of low dose effects. On the other hand, the up-regulated proteins of energy metabolism group were GAPDH, mitochondrial isoform of adenylate kinase 2 (AK2), succinate dehydrogenase Ip subunit (SDHB), and UQCRFS1 (Table 1). It is unclear, how sperm motility (Fig. 1a) and ATP (Fig. 1f) are decreased in the condition when energy metabolism proteins are increased. However, the level of expression may be suboptimal to overcome BPA-mediated toxicity, and/or the substantial increases may result in atypical functioning of these proteins.

Six cytoskeletal/structural proteins (25 %) were found to be significantly altered in 100 μM BPA-treated spermatozoa compared to those in the control cells (Table 1). The down-regulated proteins were ropporin-1 (ROPN1), ACTB, fatty acid-binding protein 9 (FABP9), and outer dense fiber protein 2 (ODF2) (Table 1). The cytoskeletal/structural proteins are important for cytoplasmic integrity, cell movement, and signal transduction maintenance. Therefore, down-regulation of these proteins could affect the cellular physiology. Clinically, down-regulation of these proteins has been found in non-motile, abnormal headed, infertile spermatozoa [18, 34]. Additionally, the functional affiliation of these proteins also has been reported to prostatic carcinoma and metastatic melanoma of the testis [35, 36]. In contrast, the up-regulated proteins were peroxisomal membrane protein 20 (PMP20) and isoaspartyl peptidase/L-asparaginase (ASRGL1) (Table 1); interestingly, neither of them had been previously described in spermatozoa. Therefore, they might be the possible biomarkers of BPA hazard assessment; however, further study needs to be conducted to strengthen this hypothesis.

Additionally, in fertility-related proteins (17 %), sperm surface protein Sp17 (SPA17) was increased by BPA exposure (100 μM), whereas prohibitin (PHB), serine/threonine-protein phosphatase PP1-alpha catalytic subunit (PPP1CA), and axonemal dynein light chain 1 (DNAL1) were decreased (Table 1). SPA17 has been reported to play a potential role in sperm-egg interactions; however, increased functionality of this protein induced immature acrosome reaction and tumor changes in cells [37], which might support our current findings. In contrast, the degraded proteins are reported to play potential roles in the regulation of sperm motility and fertilization [38, 39]. Thus, degradation of these proteins results in spermatozoa with reduced motility and viability (Fig. 1a and b). Lastly, among stress response proteins (12 %), GPX4 and PRDX5 were up-regulated, and GSTM5 was down-regulated by BPA (Table 1). It has been reported that toxic levels of ROS in mammalian spermatozoa are strongly associated with male infertility [29]. Thus, failure of the antioxidant defense system of spermatozoa to remove excess ROS levels ultimately compromises normal sperm function and fertilization. Therefore, the elevated levels of GPX4 and PRDX5 observed in the present study were assumed to offer the sperm cells a survival advantage (Fig. 1c) in a BPA-containing microenvironment. In addition, significantly increased expression of GPX4 was noted with 0.01 μM BPA treatment compared with control (Table 1), and thus, GPX4 could represent a brilliant candidate biomarker of low-dose exposure.

Our data showed that 13 of 24 differentially expressed proteins in BPA-treated spermatozoa were significantly (*p* < 0.05) correlated with five canonical pathways (Table 2). Since normal functioning of these pathways regulates cell physiology, manipulation of pathway entities definitely correlates with pathological consequences/diseases. Using Pathway Studio program, we show that the disease processes regulated by the altered proteins of these pathways were putatively involved in cancer, carcinoma, neoplasm, infertility, and so, (Fig. 4a). However, the Pathway Studio program showed the particular proteins regulated pathways, cellular processes, and diseases from entries in the PubMed and other related databases; thus, the proteins that exhibited a non-significant relationship with several pathways might also have potential clinical significance.

## Conclusions

Here, we describe the *in vitro* effects of BPA on mouse spermatozoa and the possible mechanisms of action. Additionally, we identified 24 differentially expressed proteins in spermatozoa indicative of BPA exposure and showed their affiliation with several diseases (Fig. 4b). These predicted protein markers could be used as potential candidates for developing a novel theranostic strategy for the management of BPA toxicity. Additionally, we anticipated that the proteomics and informatics tools used current study might consider for the health hazard assessment of other toxicological agents. However, further studies are required to validate preliminary finding and to better elucidate the possible clinical applications of these techniques.

## Methods

### Ethical approval

All procedures with animals were performed in accordance to protocols approved by the Institutional Animal Care and Use Committee of Chung-Ang University, Seoul, Republic of Korea.

### Media and chemicals

All chemicals and reagents were purchased from Sigma-Aldrich (St. Louis, MO, USA), unless otherwise stated. Modified Tyrode’s medium (osmolality 300 ± 20 mOsm/kg, pH 7.2 ± 0.2) was prepared freshly and used as basic medium (BM) [6, 29]. The BM was pre-incubated 1 day prior to the experiment and supplemented with bovine serum albumin (BSA; 4 mg/mL). BPA was dissolved in dimethylsulfoxide (DMSO) and added to a final concentration to treat the spermatozoa. The control spermatozoa were treated with DMSO only. An estrogenic positive control was not considered in the present study because BPA has been reported to act via other signaling pathways together with ERs; thus, it is not obvious whether BPA and natural estrogens have similar effects [40, 41].

### BPA dose selection

The exposure scheme consisted of four different doses of BPA: 0.0001, 0.01, 1, and 100 μM. The doses up to 1 μM were comparable to the acceptable human daily exposure levels [22]. It has been demonstrated that low dose of BPA (~0.01 μM) advances early embryonic development, whereas a comparatively higher dose (~100 μM) decreases the development rate of the embryo [42, 43]. More recently, genotoxic and mitogenic effects of BPA has been demonstrated at the dose of 0.01–0.1 μM, in mammary cells [44]. Therefore, range of concentrations (0.001–100 μM) was considered in current study to clarify *in vitro* adverse effect levels of BPA in spermatozoa.

### Collection and preparation of spermatozoa and their exposure to BPA

Spermatozoa were collected from sexually mature ICR male mice (Nara Biotech, Seoul, Korea) following published procedures [6, 29]. Briefly, both cauda epididymides from each mouse were collected and the associated fat was removed. The epididymides were then placed in cell culture dishes with BM containing 0.4 % BSA, and punctured using a sterile needle to release spermatozoa. The released spermatozoa were incubated for approximately 10 min with 5 % CO_2_ in air at 37 °C to facilitate dispersal. An initial experiment was designed to determine the potential of BPA to alter a) sperm motility and b) viability. For this, the spermatozoa were incubated (5 % CO_2_ in air at 37 °C) with 100 μM BPA for different periods of time (ranging from 0.5 to 8 h at 30-min intervals) to investigate the potential shift of both parameters. Finally, 6 h was identified as the minimum effective period of BPA exposure. Therefore, the sperm suspension was incubated for 6 h under the same conditions in BM supplemented with various concentrations of BPA.

### Assessment of sperm motility

Sperm motility was evaluated using computer-assisted sperm analysis (CASA) (SAIS plus version 10.1; Medical Supply, Seoul, Korea) according to described methods [6, 29]. The 10× phase contrast objective was used by the SAIS software to relay and analyze the spermatozoa. Five fields of each sample were randomly selected to evaluate the movement of at least 250 sperm.

### Hypo-osmotic swelling test (HOST)

To evaluate sperm viability and functional integrity (membrane), we used the hypo-osmotic swelling test (HOST) as described previously [45]. The sperm swelling patterns were classified broadly as viable and/or nonviable according to the WHO 2010 manual and by using the Microphot-FXA microscope (Nikon).

### Detection of LDH

To determine cytotoxicity, the CytoTox 96® assay kit (Promega, Fitchburg, WI, USA), which is based on the colorimetric detection of LDH, was used according to the method described previously [6, 29]. The LDH activity was measured as the absorbance at 490 nm by using a luminometer (GEMINI EM, Molecular Devices Corporation) and was calculated using the SoftMax Pro 5 software. Activity is reported as the ratio of the fluorescence of BPA-treated samples to that of the control.

### Detection of mitochondrial activity

Mitochondrial membrane potential, defined as mitochondrial activity, was measured by rhodamine 123 (R123) staining according to the manufacturer’s directions and the described method [45]. The samples were analyzed by flow cytometry (Becton Dickinson, Franklin Lakes, NJ, USA) with an excitation wavelength of 488 nm and an emission wavelength of 525 nm. Ten thousand cells in each sample were considered to obtain R123 signal, and the signal was analyzed using the CellQuest software (Becton Dickinson). Mitochondrial activity was reported as the fluorescence of the BPA-treated samples compared with that of the control.

### Detection of [ATP]_i_ levels

[ATP]_i_ was detected using an ATP Bioluminescence Assay Kit (CLS II; Roche Molecular Biochemicals, Mannheim, Germany) according to the described method [29]. The luminescence signal was detected using a Microplate Multimode Reader (GloMax®-Multi; Promega, Madison, WI, USA). The [ATP]_i_ is reported as the ATP (signal) of the BPA-treated samples compared with that of the control.

### Preparation of spermatozoa for proteomic experiments

The BPA-treated and control spermatozoa after 6 h of incubation were washed twice by centrifugation (100 × *g* for 2.5 min) at room temperature (RT), re-suspended in BM, and allowed to swim-up at 37 °C for an additional 15 min. Swim-up was performed to separate the motile sperm fraction from immature spermatozoa and somatic cells [46–48]. The motile sperm fraction was then carefully collected, and the samples were checked for the nonappearance of immature spermatozoa and somatic cells using light microscopy and Hoechst staining (Additional file 2: Figure S3).

### 2-DE and gel-image analysis

To extract proteins from the spermatozoa, 50 × 10^6^ cells were incubated in rehydration buffer for 1 h at 4 °C [17, 18, 48]. Then, 250 μg aliquots of sperm protein were placed in 450 μL of rehydration buffer in a rehydration tray with 24 cm-long NL Immobiline DryStrips (pH 3–11; Amersham, Piscataway, NJ, USA) for 12 h at 4 °C. The first-dimension electrophoresis (1-DE) was performed using an IPGphor IEF apparatus, and the strips were focused according to previously described settings [17, 18, 48]. After iso-electrofocusing, the strips were equilibrated for 15 min at RT by using equilibration buffer A, whereas the second equilibration was performed using equilibration buffer B [17, 18, 48]. Afterward, 2-DE was carried out on 12.5 % (w/v) SDS-PAGE gels with the strips at 100 V for 1 h and then at 500 V until the bromophenol blue front began to migrate off the gels. The gels were silver-stained for image analysis according to the manufacturer’s instructions (Amersham, Piscataway, NJ, USA). The silver-stain was used to conduct the present study because this method is highly sensitive, capable of detecting minimal variants of protein in the gel, and is compatible with downstream processing, such as mass spectrometry [18]. The gels were scanned using a high-resolution GS-800 calibrated scanner (Bio-Rad, Hercules, CA, USA). Detected spots were matched and analyzed by comparing the gels from spermatozoa treated with BPA and the control using PDQuest 8.0 software (Bio-Rad, Hercules, CA, USA). Finally, the spot density was calculated and normalized as the ratio of the spot on the BPA-treated (spermatozoa) gel to that on the control gel.

### Protein identification

#### In-gel digestion

Proteins were subjected to in-gel trypsin digestion according to the protocol established previously [17, 18]. Excised gel spots were destained with 100 μL of destain solution (30 mM potassium ferricyanide, 100 mM sodium thiosulfate) by shaking for 5 min. Then, the gel spots were incubated with 200 mM ammonium bicarbonate for 20 min. The gel pieces were dehydrated with 100 μL of acetonitrile and dried in a vacuum centrifuge. The above procedure was repeated thrice. Then, the dried gel pieces were rehydrated with 20 μL of 50 mM ammonium bicarbonate containing 0.2 μg of modified trypsin for 45 min on ice. Seventy microliters of 50 mM ammonium bicarbonate was added after removal of solution. Digestion was performed overnight at 37 °C. The peptide solution was desalted using a C18 nano column (homemade, Waters Corp, Milford, MA, USA).

#### Desalting and concentration

The custom-made chromatographic columns were used for desalting and concentration of the peptide mixture. A column consisting of 100–300 nL of Poros reverse phase R2 material (PerSeptive Biosystems, Framingham, MA, USA) was packed in a constricted GELoader tip (Eppendorf, Hamburg, Germany). The liquid was forced gently into the column using a 10-mL syringe. Thirty microliters of the peptide mixture from the digestion supernatant was diluted with 30 μL of 5 % formic acid, loaded onto the column, and washed with 30 μL of 5 % formic acid. Peptides were eluted with 1.5 μL of 50 % methanol/49 % H2O/1 % formic acid directly into a precoated borosilicate nanoelectrospray needle (New Objective, Woburn, MA, USA) for analysis by tandem mass spectrometry (MS/MS).

#### ESI-MS/MS

MS/MS of peptides generated by in-gel digestion was performed by nano-ESI on a MicroQ-TOF III mass spectrometer (Bruker Daltonics, Germany) at RT. A potential of 1 kV was applied to the precoated borosilicate nanoelectrospray needles (EconoTipTM, New Objective) in the ion source and combined with a nitrogen back-pressure of 0–5 psi to produce a stable flow rate (10–30 nL/min). The cone voltage was 800 V. The quadrupole analyzer was used to select precursor ions for fragmentation in the hexapole collision cell. Product ions were analyzed using an orthogonal TOF analyzer, fitted with a reflector, a micro-channel plate detector, and a time-to-digital converter. The data were processed using a peptide sequence system.

### Database search

An MS/MS ion search was allocated as the ion search preference in the MASCOT software (Matrix 20 Science, Boston, MA, USA). Peptide fragment files were obtained from the peptide peaks in ESI-MS by ESI-MS/MS. Trypsin was selected as the enzyme with one potentially missed cleavage site. ESI-QTOF was selected as the instrument type. The peptide fragments were searched based on the database using the MASCOT (v2.4, Matrix Science) and FASTA search engine, and the search was limited to *Mus musculus* taxonomy in NCBInr, UniprotKB/TrEMBL and UniprotKB/Swissprot database. The mass tolerance was set at ± 1 and ± 0.6 Da for the peptides and fragments, respectively. High-scores were defined as those above the default significance threshold in MASCOT (*p* < 0.05, peptide score, >30).

### Western blotting

Western blot analysis was performed as previously described [6, 29]. The antibodies were diluted in 3 % blocking agent: anti-phospho-p38 MAPK Antibody (1:1000, Cell Signaling, Danvers, MA), Anti-PI3K p85 (phosphor Y607) antibody (1:1000, Abcam, Cambridge, UK), anti-phospho-PKA substrate antibody (1:10,000; Cell Signaling Technology, MA, USA), anti-phosphotyrosine antibody (PY20, 1:2,500, Abcam), anti- GAPDH antibody (1:1000, Abcam), anti-UQCRFS1 antibody (1:15,000, LSBio, Inc.), anti- PRDX5 antibody (1:2000, Abcam), anti-GPX4 antibody (1:15,000, Abcam), anti-ACTB antibody (1:500, Abcam), and anti-GSTM5 antibody (1:5,000, Abcam). Anti-α-tubulin mouse antibody (1:1000, Abcam) was used as the loading control for all western blots. The proteins on the membranes were detected with by an enhanced chemiluminescence (ECL) technique using ECL detection reagents.

### Signaling pathways

Pathway Studio (v 9.0, Aridane Genomics, Rockville, MD, USA) program was used to predict the signaling pathways and biological functions of BPA-mediated differentially expressed (>2-fold; *p* < 0.05) proteins according to previously described procedure [29, 48]. Briefly, differentially expressed proteins were entered into the Pathway Studio in order to determine the significantly matching pathways for each protein (*p* < 0.05). To fulfill the additional objectives, groups of proteins that significantly related to particular pathways were subject to a MedScan Reader (v5.0) search, and their functional affiliation with disease processes were predicted using Pathway Studio. The signaling pathways and biological functions were confirmed by the PubMed Medline hyperlink that was embedded in each node.

### Statistical analysis

The data were analyzed by one-way ANOVA using SPSS program (v. 18.0, Chicago, IL, USA), and Tukey’s test was used to locate differences. *P* values of <0.05 were considered statistically significant. All data are expressed as mean ± SEM. The probabilities of the signaling pathways were determined using the Fisher’s exact test (*p* < 0.05).

## Additional files

Additional file 1: Table S1. Peptide sequence, matches peptides, and searching engines of identified proteins. (PDF 99 kb) Additional file 2: Figure S1. Validation of the 2-DE results by western blot analysis. (**A**) The bars represent the densities of particular proteins bands, such as GAPDH, UQCRFS1, PRDX5, GPX4, ACTB, and GSTM5. Data are presented as mean ± SEM (*n* = 3). Values with different superscript characters (A,B,C) indicate significant differences between the control and treatment samples as determined by one-way ANOVA (*P* < 0.05). (**B**) Representative western blot image of GAPDH, UQCRFS1, PRDX5, GPX4, ACTB, and GSTM5 probed with the respective antibodies. **Figure S2.** Effect of BPA on tyrosine phosphorylation status of spermatozoa. (**A**) Density of phosphotyrosine proteins in control and BPA-treated samples. Data are the mean of four replicates ± SEM. Values with different upper and lower case letters (A,B,C,a,b,c) are significantly different between control and treatment groups by one-way ANOVA. (**B**) Tyrosine-phosphorylated proteins were probed with PY 20. Lane 1: control; lane 2: 0.0001 μM BPA; lane 3: 0.01 μM BPA; lane 4: 1 μM BPA; and lane 5: 100 μM BPA. **Figure S3.** Validation of separation technique of spermatozoa for proteomic analysis. Spermatozoa examined for the non-appearance of immature sperm cells and somatic cells using microscopy. (**A**) Differential interference contrast microscopic (DIC) image of spermatozoa used for proteomic analyses. (**B**) Image of hoechst staining spermatozoa. (**C**) Merged image. (PDF 936 kb)
