# Supplementary material for: A novel approach to assessing bisphenol-A hazards using an in vitro model system
Source: BMC Genomics. 2016 Aug 9;17:577. doi: 10.1186/s12864-016-2979-5 (PMC4977886; doi:10.1186/s12864-016-2979-5)
Supplement: Additional file 1: Table S1. — Peptide sequence, matches peptides, and searching engines of identified proteins. (PDF 99 kb) [file 12864_2016_2979_MOESM1_ESM.pdf]

Additional file 1. Supplementary table 1

Table S1: Peptide sequence, matches peptides, and searching engines of identified proteins.

Symbol	Peptide	Matched peptide	Search engine (database)
ATP5H	R.LASLSEKPPAIDWAYYR.A	606.6	MASCOT (NCBInr)
ATP5O	P.PVQVYGIEGR.Y	528.3	FASTA (UniProtKB)
HADHA	E.YLEEVAVNFAK.G	813.4	FASTA (UniProtKB)
COX6A1	K.ALTYFVALPGVGVSMLNVFLK.S	752.4	MASCOT (SwissProt)
PGAM2	R.ALPFWNEEIAPK.I	707.8	MASCOT (SwissProt)
OXCT2A	K.GLTLVELWEGSSVDDIK.A	930.9	MASCOT (SwissProt)
ISOC2A	S.SILFLCDLQEK.F	953.3	FASTA (UniProtKB)
GAPDH	K.LVAWYDNEYGYSNR.V	875.4	MASCOT (SwissProt)
AK2	K.LVSDEMVVELIEK.N<br>K.LDSVIEFSIQDSLLIR.R	760.4, 924.5	MASCOT (NCBInr)
SDHB	K.LQDPFSVYR.C	562.8	FASTA

			(UniProtKB)
UQCRFS1	A.TTTVGVAYAAK.N	683.3	FASTA (UniProtKB)
ROPN1	R.FTEEIEWLK.F<br>R.LIIHADELAQMWK.V<br>K.IVCEVLSSDHDGGPPR.I<br>R.MLNYIEQEVIGPDGLIK.V	528.6, 579.9,597.8, 974.5	MASCOT (NCBInr)
ACTB	R.AVFPSIVGRPR.Q<br>K.SYELPDGQVITIGNER.F	599.8, 895.9	MASCOT (SwissProt)
FABP9	M.IEPFLGTWK.L<br>K.SLITFEGGSMIQVQK.W	619.3, 827.4	MASCOT (SwissProt)
ODF2	R.QFQSQLADLQQLPDILK.I	993.0	MASCOT (NCBInr)
PMP20	K.ATDLLLDDSLVSLFGNR.R	924.9	MASCOT (NCBInr)
ASRGL1	K.TVEEAAQLALDYMK.S	791.3	MASCOT (SwissProt)

PHB	K.AAELIANSLATAGDGLIELR.K<br>R.FDAGELITQR.E	575.3, 999.5	MASCOT<br>(NCBInr)
PPP1CA	R.EIFLSQPILLELEAPLK.I	977.5	MASCOT<br>(SwissProt)
DNAL1	K.LYAQIPPIEK.M	586.3	FASTA<br>(UniProtKB)
SPA17	R.IPQGFGNLLEGLTR.E	757.9	MASCOT<br>(NCBInr)
GSTM5	K.LTFVDFLTYDVLDQNR.M	980.0	MASCOT<br>(NCBInr)
GPX4	N.YTQLVDLHAR.Y	548.9	FASTA<br>(UniProtKB)
PRDX5	R.LLADPTGAFGK.A	545.2	FASTA<br>(UniProtKB)
