# Supplementary material for: A novel approach to assessing bisphenol-A hazards using an in vitro model system
Source: BMC Genomics. 2016 Aug 9;17:577. doi: 10.1186/s12864-016-2979-5 (PMC4977886; doi:10.1186/s12864-016-2979-5)
Supplement: Additional file 2: Figure S1. — Validation of the 2-DE results by western blot analysis. (A) The bars represent the densities of particular proteins bands, such as GAPDH, UQCRFS1, PRDX5, GPX4, ACTB, and GSTM5. Data are presented as mean ± SEM (n = 3). Values with different superscript characters (A,B,C) indicate significant differences between the control and treatment samples as determined by one-way ANOVA (P < 0.05). (B) Representative western blot image of GAPDH, UQCRFS1, PRDX5, GPX4, ACTB, and GSTM5 probed with the respective antibodies. Figure S2. Effect of BPA on tyrosine phosphorylation status of spermatozoa. (A) Density of phosphotyrosine proteins in control and BPA-treated samples. Data are the mean of four replicates ± SEM. Values with different upper and lower case letters (A,B,C,a,b,c) are significantly different between control and treatment groups by one-way ANOVA. (B) Tyrosine-phosphorylated proteins were probed with PY 20. Lane 1: control; lane 2: 0.0001 μM BPA; lane 3: 0.01 μM BPA; lane 4: 1 μM BPA; and lane 5: 100 μM BPA. Figure S3. Validation of separation technique of spermatozoa for proteomic analysis. Spermatozoa examined for the non-appearance of immature sperm cells and somatic cells using microscopy. (A) Differential interference contrast microscopic (DIC) image of spermatozoa used for proteomic analyses. (B) Image of hoechst staining spermatozoa. (C) Merged image. (PDF 936 kb) [file 12864_2016_2979_MOESM2_ESM.pdf]

Additional file 2. Supplementary figures 1 to 3

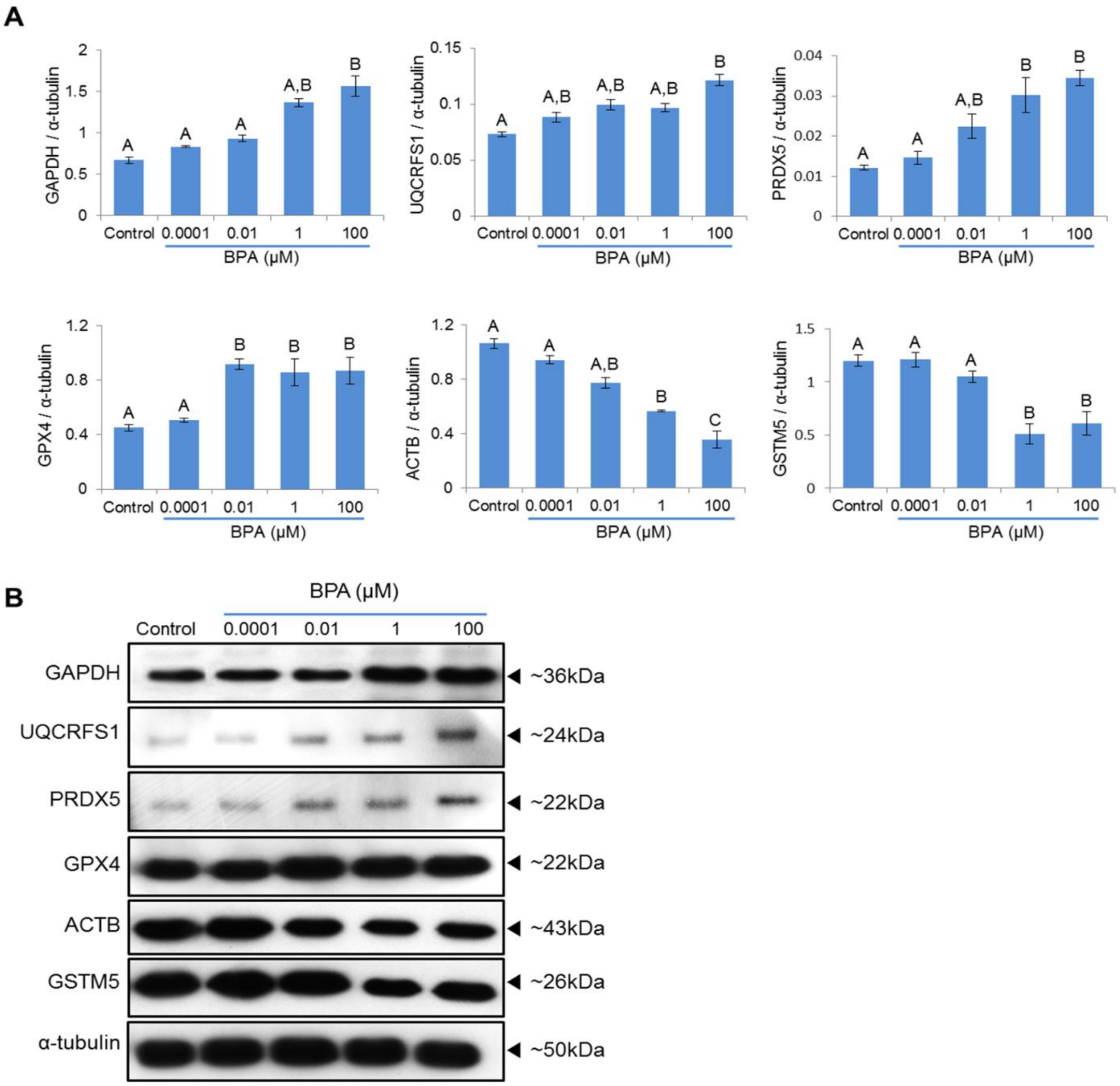

Fig. S1 Validation of the 2-DE results by western blot analysis. (**A**) The bars represent the densities of particular proteins bands, such as GAPDH, UQCRFS1, PRDX5, GPX4, ACTB, and GSTM5. Data are presented as mean ± SEM (n=3). Values with different superscript characters ([A,B,C]) indicate significant differences between the control and treatment samples as determined by one-way ANOVA ($P < 0.05$). (**B**) Representative western blot image of GAPDH, UQCRFS1, PRDX5, GPX4, ACTB, and GSTM5 probed with the respective antibodies.

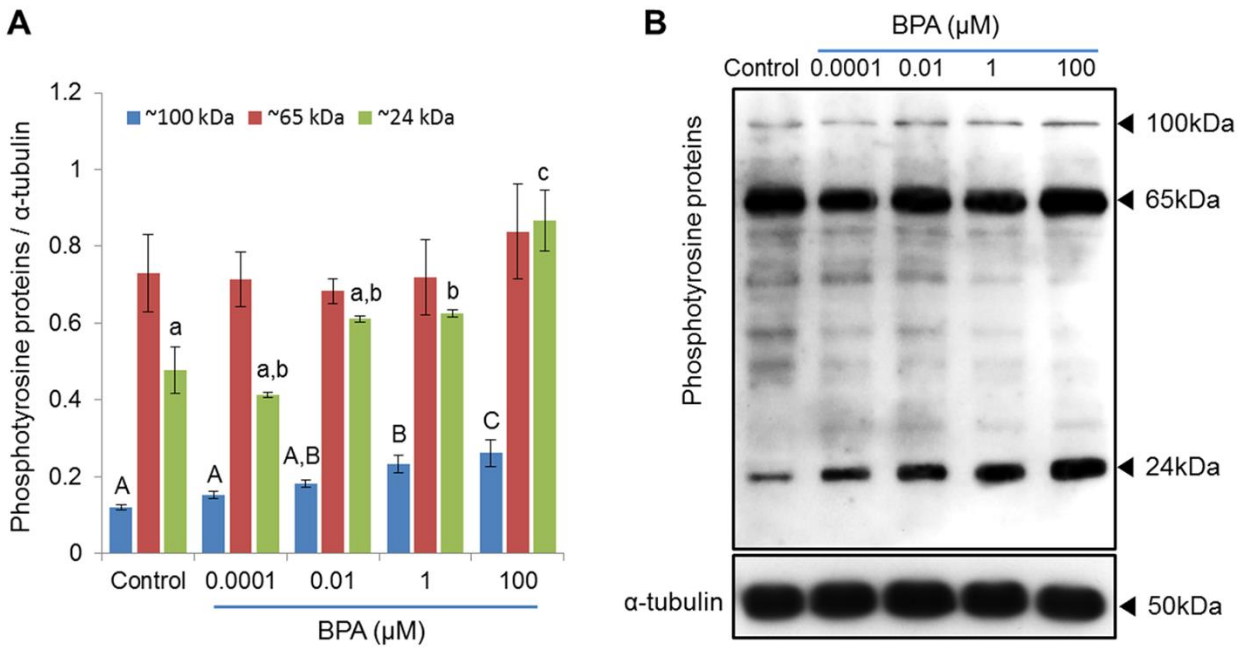

Fig. S2 Effect of BPA on tyrosine phosphorylation status of spermatozoa. (**A**) Density of phosphotyrosine proteins in control and BPA-treated samples. Data are the mean of four replicates ± SEM. Values with different upper and lower case letters ([A,B,C,a,b,c]) are significantly different between control and treatment groups by one-way ANOVA. (**B**) Tyrosine-phosphorylated proteins were probed with PY 20. Lane 1: control; lane 2: 0.0001 µM BPA; lane 3: 0.01 µM BPA; lane 4: 1 µM BPA; and lane 5: 100 µM BPA.

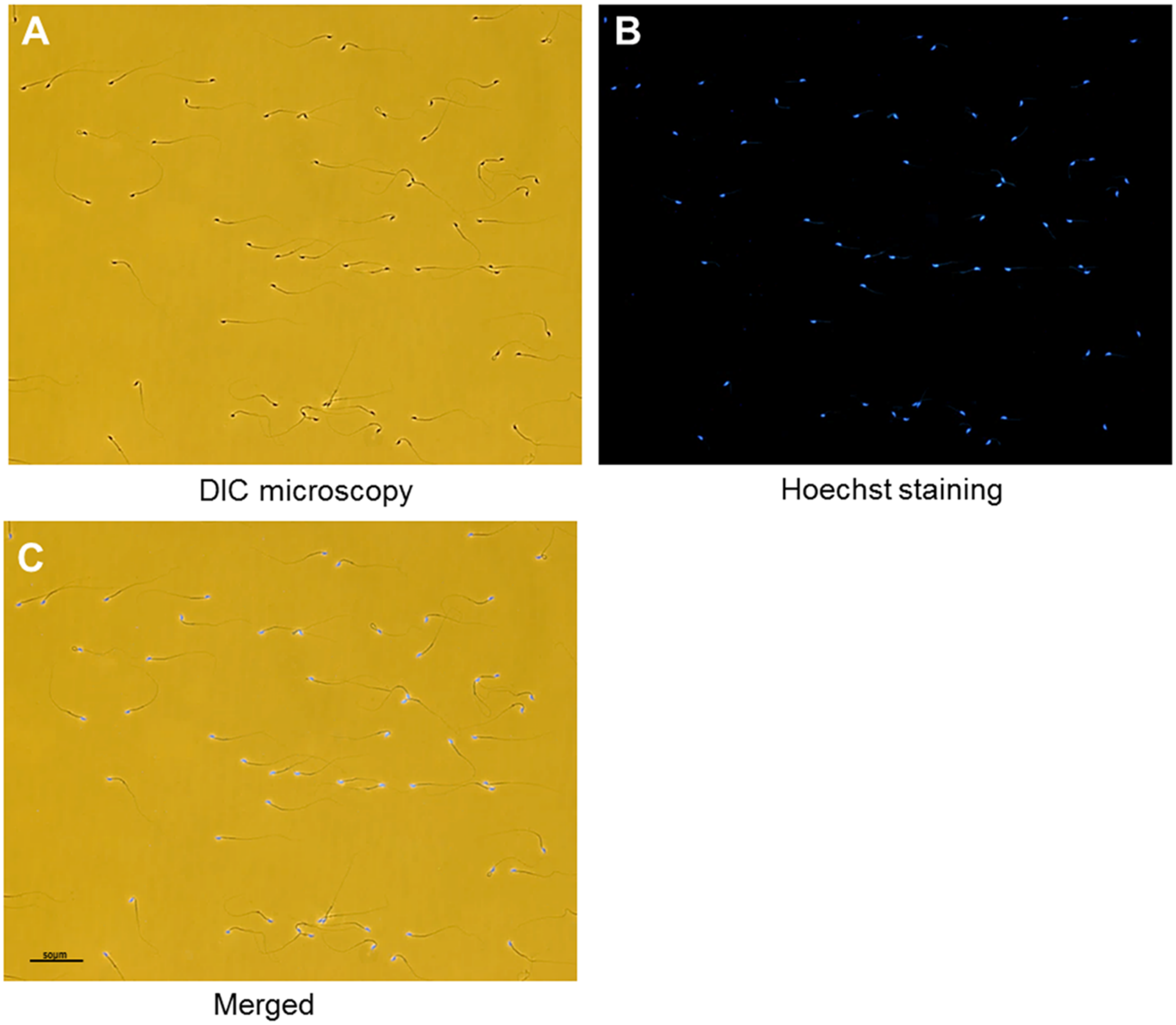

Fig. S3 Validation of separation technique of spermatozoa for proteomic analysis. Spermatozoa examined for the non-appearance of immature sperm cells and somatic cells using microscopy. (**A**) Differential interference contrast microscopic (DIC) image of spermatozoa used for proteomic analyses. (**B**) Image of hoechst staining spermatozoa. (**C**) Merged image.
